# Iridectomy as a Therapeutic Measure

**Published:** 1874-11

**Authors:** Swan M. Burnett

**Affiliations:** Knoxville, Tenn.


Vol. IV.—NOVEMBER, 1874.—No. 11.

### Original Communications.

### IRIDECTOMY AS A THERAPEUTIC MEASURE.

BY SWAN M. BURNETT, M.D., KNOXVILLE, TENN.

[*Read before the Knox County Section of the East Tennessee Medical Society.*]

The operation of iridectomy has been used for the purpose of making an artificial pupil, since an early period in the history of ophthalmic surgery. Its use, however, as a therapeutic means, owes its origin entirely to the great genius of von Graefe. Although he made valuable discoveries, and contributions to our knowledge, in almost every department of the science to which he devoted the energies of his life, his name will probably, in after times, be most closely associated with the operation of iridectomy as a cure for glaucoma and kindred diseases.

It is not our purpose here to enter into a consideration of glaucoma proper, but simply to point out the application of iridectomy as a means of cure in some other, but similar conditions of the eye, as illustrated by two cases that have occurred in my practice within the last few months; and more particularly do I wish to call the attention of the general practitioner to the important fact, that in all the cases the symptom which called most urgently for relief was that of pain, and pain of such character as would, upon a superficial examination, be deemed as simply neuralgic, and distinct from any eye affection.

The first case was that of a married lady, aged 21. Some six years ago she had an inflammation of the right eye, accompanied with excessive pain. Under treatment it subsided, but has returned at intervals, up to this time. More latterly, however, the attacks have been more of the character of neuralgia of the right side of the head and face. The attacks generally pass off in the course of three or four days. An examination showed a synechia posterior complete, except at the upper one-fifth of the pupil. Shape of pupil, in consequence of synechia, slit-shaped and vertical. Pupil was clear, but behind the iris and posterior to the lens, on the nasal side, there was a whitish looking substance, such as we see in exudation from the choroid. She has at times been troubled with flashes of light. Can barely count fingers at a few feet. Opthalmoscopic examination not possible. I could not make any promises as to improvement in vision by means of an operation, because it was not possible to estimate the extent of the intra-ocular exudation; but, according to the experience of Graefe, an excision of a portion of the iris would relieve the ciliary neuralgia, and put a stop to any choroidal inflammation that might still be present, and would, upon the whole, put the eye in much better condition and lessen immensely the chances of a panophthalmitis, or glancoma.

These facts were laid before her, and, without hesitation, she assented to an operation. She was willing to risk anything that offered any hope of relief.

Accordingly, I made an irridectomy outwards, removing about one-fifth of the iris, without anesthesia. She recovered from the operation well, suffering no pain except a little on the third day after the operation. She gradually improved from that date, and at the last time I saw her she had had no return of pain, and her vision had so improved that she was able to decipher the largest print. It appeared, as I found after the operation, that I was fortunate in selecting the place for the operation, for the exudation was confined entirely to the nasal side, reaching to about the middle of the lens, leaving the temporal half of the vitreous clear.

About four months after the operation, an incident occurred which has an interest of its own, and which, as it may have some connection with the other features of the case, ought perhaps to be mentioned here.

She awoke one morning with an intense pain over both eyes, and affecting more or less the whole head. This continued all the day, and into the next night. The next morning she found she was unable to raise the lid of the right eye. I found a complete paralysis of all the muscles supplied by the third pair—that is, the levator palpebrarum, and all the ocular muscles except the rectus externus and superior oblique. The eyeball was consequently immovable in all directions except outwards, by means of the external rectus, and downwards and outwards, by the conjoint action of the external rectus with the superior oblique. There must also have been a paralysis of the accommodative muscle; for, although before the attack she had been seeing quite well, now she was barely able to discern the forms of objects. Under heavy doses of iodide of potassium and the application of the induced current of electricity over the closed lid, she recovered completely in the course of three weeks.

The result in this case was much better than was hoped for. Improvement in vision followed which was not anticipated.

The second case was Miss W., aged 21. The history she gave, when she presented herself to me was, that five months previous she had an attack of sore eyes, and that the right eye had bursted and the water ran out. Soon after, pain set in in the right brow and right side of the head. This pain has continued until now, and at times has been most excruciating; it is worst at night. Ten days ago noticed some blue lumps on the upper portion of the eyeball, which have been gradually enlarging ever since.

On examination, I found an opacity of the cornea a little to the temporal side and slightly above the centre—the cicatrix of a perforation, and in this cicatrix the iris was entangled; anterior chamber moderately deep; pupil dilated moderately; commencing ciliary staphyloma at four distinct points; tension considerably increased; suffers much with ciliary neuralgia; sees latterly quite often flashes of light; can count fingers at three feet only, yet the opacity does not cover the pupil. I can see, but not in detail, the fuudus with the ophthalmoscope—turbidity of the vitreous.

Here was marked increase of intra-ocular tension, with a tendency to staphyloma, which, if not checked, would lead to most pernicious results in the form of glaucoma, and to a hideous deformity in the shape of ciliary staphyloma. These and the intense pain called loudly for some interference.

The only thing that offered any hope of relief was an iridectomy, which, by lessening the intra-ocular pressure and removing the source of irritation in the anterior synechia, would give relief both to the glaucomatous and staphylomatous symptoms and the intense ciliary neuralgia. I determined, therefore, to remove a large portion of the iris, including that part entangled in the corneal cicatrix. An incision was accordingly made a little anterior to the sclerotico-corneal junction on the temporal side, and the iris gently drawn out; but just as it was clipped close to the globe, it was discovered that it had given way from its attachment on the opposite side of the eye. Nothing now remained but to take away the whole iris, which was accordingly done. Much hemorrhage followed. The eye was bandaged and the patient was put to bed. That night she suffered no pain. The next morning the staphylomatous bulgings were level with the sclerotic and the tension was normal; large clot of blood in the anterior chamber. The case progressed well until the fourth day, when there was some return of pain, which yielded to opiates. Twice after that she had pain which lasted a few hours, but for the last two months has suffered no inconvenience from it. At last examination, some three months after the operation, the staphyloma had not reappeared, but its former site was marked by three or four bluish places on the sclerotic. Vision gradually improving.

An interesting feature in this case is the rupture of the iris from its ciliary attachment under the gentlest traction.

I find a cause for this in the softening of its tissue at that place from the *choroditis anterior*, which was evidently present. The iris itself did not seem to be friable, but it gave way very close up to its attachments, so that the edge of the lens was readily seen in ophthalmoscopic examination, and a distance of two milimeters between the edge of the lens and the ciliary region. The opacity of the cornea was, at last accounts, gradually clearing up, but there is no hopes that it will all be absorbed.

I had thought to give you some other cases, but these are sufficient to show you the immense therapeutic powers of the operation of iridectomy. I cannot here go into a discussion of the *modus medendi*, for opinions are divided upon it to such an extent as to make a choice between them well-nigh impossible. But the empirical fact we have, and that is sufficient for our present purpose.

It will be noticed that relief of pain, a neuralgia, was one of the most marked effects of the operation, and what I would impress upon the members of this Society is, that when they have a severe, obstinate orbital neuralgia, with an antecedent history of ophthalmia, to search for an adhesion of the iris, and if it is found, you will then have a sufficient cause for your complaint. The only thing that then remains to be done is to do away with the source of irritation, and this can best be accomplished by excising a piece of the iris. In making the operation of iridectomy for therapeutic purposes, it should always be large; whereas, if it is for visual purposes alone, the smaller it is the better. These are only some of the uses the operation may be put to for the cure of disease in the eye. In nearly all cases of intra-ocular inflammation, and in fact, obstinate inflammation in any part of the eyeball, it has been used, and with benefit, and its field of usefulness is constantly extending with our increase of clinical experience.

